# Medical Colleges

**Published:** 1884-10

**Authors:** 


### Medical Colleges.

Before this number reaches our readers, the Medical Colleges of this city will be in full operation. The Buffalo Medical College opened its session September 25th, with an introductory address by Dr. J. W. Keene on "The Relations of the Teacher and Student of Medicine." We need not say that whatever production comes from so cultured a mind as that of the lecturer on that occasion, will furnish valuable pabulum for thought and reflection to students, as well as practitioners of medicine.

The Niagara Medical College opened its course October 1st, with an address by Prof. W. S. Tremaine on "Medical Education." The well-known ability of the distinguished professor of surgery was abundantly shown on the occasion, and we voice the sentiment universally expressed by his hearers, when we say that the Niagara Medical College shows its inherent strength when one of its faculty utters sentiments on this important subject, which echo the advanced thought of the profession of this country.

On account of the failure to complete the college building in time for the opening of the session, the lectures will be given at the Young Men's Christian Association Building on Mohawk street. It is expected that the new building will be ready for occupancy by December 1st, when this school will possess ample accommodations and most conveniently arranged apartments for its work.

---

We direct the attention of our readers to the interesting remarks of Prof. Stockton of the Niagara Medical College, made before the Buffalo Medical and Surgical Association, in reference to artificial alimentation in infants, which we publish in this number of the Journal. The subject is of the greatest importance, and is also attended with the greatest difficulty; and, until this solution of the problem was reached, we have been at a loss to know how to treat successfully the derangements due to defective alimentation so frequently met with in our large cities during the heated term. It will be noticed that the milk diet is emphasized as the essential nutriment for infants. The question has always been how to adapt cow's milk to the child's powers of digestion and assimilation. We fully endorse the principle enunciated by the speaker that mal-nutrition and enfeebled constitutions result from the use of cereals and prepared foods. A healthy balance in the nutrition of the organization only results from a diet of milk, and all efforts to substitute other foods inevitably result in failure. The preparations of milk, as directed by Dr. Stockton, are based on sound physiological principles, and practically are found beneficial in their effects. Their introduction marks an important advance in infant dietetics, and furnishes the profession with an aliment for successful use in the gastro-intestinal diseases, which are so prevalent, and difficult of treatment, during the heated term. We have found, in our professional experience, the peptonized, and pancreatized milk to be adapted very generally to digestive derangements of adults as well as of children. The specific directions for their preparation are important and should be strictly observed. We venture, also, to direct special attention to the remarks of Dr. Cronyn that the part of the alimentary tract affected should be carefully diagnosed. This will be a guide to the selection of the preparation of milk to be used, whether peptonized or pancreatized, the former being indicated in gastric indigestion and the latter in duodenal and intestinal derangements. It is also an interesting study to observe how many complications are the direct result of gastro-intestinal derangements, and how much they are under the influence of proper food. These diseases also suggest the extensive reflex nervous influences prevailing in the growing organization of children. How to maintain a healthy equilibrium of the nervous and vascular forces, so readily disturbed in the young and tender child, is a question which turns more upon the selection of proper food than upon medication. These questions in pediatrics open a wide field for study and investigation. For derangements depending on malnutrition, as well as for disturbances of the equilibrium of the vital forces of the body, the principles laid down by Dr. Stockton seem to offer a satisfactory solution. We hope the profession will give these subjects the attention their importance demands. We believe the large mortality resulting from gastrointestinal diseases, due to prolonged heat and improper food, will be very much lessened thereby.

Parke, Davis & Co. have furnished us a convenient and tastefully arrangee case, enclosing Oliver's urinary test papers, with instructions for their use. They are designed for the clinical examination of urine at the bed-side. The case contains litmus paper, which serves as a test for acidity or alkalinity of the secretion; as tests for albumen, the case includes four of the recently introduced reagents: 1, Picric acid; 2, Potassio-mer curic iodide; 3, Potassium-ferro-cyanide; 4, Sodium tungstate, all of which are to be used in connection with citric acid. As tests for sugar, the series of test papers includes, first, a modification of the usual copper test, and, second, picric acid in combination with carbonate of sodium. For use by the physician, especially in the country, where chemicals are not easy of access, this compact arrangement meets a want which has been long felt. We have used them with success and satisfaction. The case contains, also, a graduated pipette and specific gravity beads, with full directions for their use. We direct attention to this case with a view to commend them to many in the profession who have neither the time nor the taste to convert their office into a chemical laboratory for analytical investigations, and who need just such a compact test case to take with them in their daily practice.

---

Dr. Ferguson, of Toronto, Canada, reports (*New York Medical Journal*) a case of post partum synovitis. The patient, a primipara, suffered from septicæmia for several days, and in four weeks after delivery was able to be up. Two weeks after pains began to attack the various joints, attended with tenderness, slightly swollen, faintly reddened and painful on motion. No abscesses formed during the progress of the case, neither was there much of a rise of temperature, or pulse above the normal. The tenderness and swelling of each joint subsided in about four days to a week. To quote: "Barwell, in his work on the diseases of the joints, mentions that he has met with, altogether, four cases of post partum synovitis; and, from his description, they pursued a course somewhat like any case. Some infection is taken up from the utero-vaginal tract, which acts injuriously on the joints, giving rise to metastatic synovitis." Salicine and salicylic acid were used without benefit. This would not point toward rheumatism as a cause. She improved steadily upon Barwell's plan of treatment: fifteen to twenty grains of sulpho-carbolate of sodium with two grains of quinine in half an ounce of camphor water three times a day.

---

DR. THEOBOLD, of Baltimore, reports the removal, by a physician, from the nose of a lady, of a suspicious looking mass which had apparently sloughed off from its former attachments the supposed neoplasm being regarded as probably of a malignant character. It was examined with the microscope, and at one of the meetings of a Baltimore society, its histological characteristics, together with the clinical facts, were duly presented. An animated discussion followed, and more than one hypothesis was advanced to account for the unusual features of the case. During the heat of this debate, an inquisitive individual inspected the supposed tumor with more care than others had done, and announced that it was a harmless specimen of the "bi-valve" order, familiarly known as the *oyster*. It was half digested and had probably became lodged in the nose during a previous spell of vomiting. This is a forcible illustration of discussions, "long, labored and loud," too often held over imaginary or misconstrued "facts."

---

DR. W. W. SEELY, of Cincinnati, has found a new use for jequirity, the remedy which is becoming so popular in the treatment of trachoma of the conjunctiva. In a paper read before the American Otological Society, at its recent meeting, he states that he has, for the past year or two, treated certain long-standing cases of purulent inflammation of the middle ear by exciting an additional substantive inflammation with jequirity, using a small quantity of the preparation made for the eye. He thought such inoculation capable of good results under certain conditions, viz., when there was extensive destruction of the membrana tympani with a great amount of thickening of the mucous membrane of the tympanic cavity, rendering other plans futile, and where the Eustachian tube was patulous, so as to regulate the inflammation.

---

LAWSON TAIT pays the following tribute to American medical schools: "In my early days the medical education of a British youth was not considered complete unless he had made a tour of the schools of France and Germany, and, like others, I felt of myself as was said of Proteus:

> ''Twould be a great impeachment to his age
> In having known no travel in his youth.'

But I wish now that the time and money spent had been directed to the western instead of the eastern continent, and I now venture to predict that ere long it will be to the medical schools of America rather than to those of Europe that our students will travel, as did the apprentices of old before they settled down to the serious exercise of their craft."

---

ANTIPYRIN is a new anti-pyretic and is recommended as a substitute for quinine. It is a white crystalline powder, with a slight aromatic odor and somewhat bitter taste. It is soluble in water, and the dose as an anti-pyretic is fifteen to thirty grains. Drs. Penzoldt and Sartorius, who have made numerous trials of the drug in diseases of children, regard it as a very effectual remedy in the pyretic diseases of childhood. It reduces the temperature several degrees, but not the frequency of the pulse to a corresponding degree, and the only disturbance it causes is, occasionally, vomiting. They give it in three doses, at an interval of an hour, each dose consisting of as many decigrams (1½ grs.) as the child has lived years.

Lawson Tait, the eminent surgeon and ovariotomist of Birmingham, England, is now visiting in the United States, and is most warmly received by the profession wherever he goes. His success and boldness in abdominal surgery have given him the attention of the whole medical world, and his independent and fearless criticism has been severely felt, not less in high and honored, than in more humble professional circles. His address before the Canadian Medical Association, at its meeting in Montreal, August 25th, is an emphatic and pronounced epitome of his views on abdominal surgery, and of his opinions of methods and men connected with its history. Every surgeon should read this address.

---

The American Opthalmological and the American Otological Societies held most successful meetings at the Catskills, in July. The various sections of the United States were well represented, and the papers and discussions were highly interesting. The excellent work done by each of these organizations, and the zeal with which members pursue it, are equal to that of any medical or surgical society with which we are acquainted. The profession is greatly enriched by such labors, and such careful investigations.

---

Dr. Geo. H. Kidd, in his address in obstetric medicine before the British Medical Association at Belfast, took puerperal fever for the subject of discussion. He takes the ground opposed to Schroeder, as announced in the nine propositions, that puerperal fever is nothing but poisoning with septic matter from the genital organs. Dr. Kidd believes that a large number of cases occurring in a community during a special time may be called an epidemic, and that epidemic puerperal fever is a specific disease.

---

Prof. W. H. Heath, of the Niagara Medical College, sailed for Europe Sept. 27th, to be absent about two months, for the benefit of his health. His position in the college will be temporarily filled, while the duties of hospital marine service of this city, which devolved upon him, will be performed by Dr. Bennett, transferred to this city for that purpose. We wish our esteemed colleague *bon voyage*.

---

The effects of operation for lacerated cervix, upon dilatation of the os, at subsequent labors, has been a fruitful source of speculation in the past. The observations of such operators as Skene, Hanks, Lee, Pallen, Barr, Gittings, Montgomery and Goodell prove that a large majority of the cases operated upon do not have a re-laceration at subsequent deliveries.

---

Jerome A. Anderson, M. D., of San Francisco, in the *American Journal of Obstetrics*, advances a new theory of fœtal nutrition. He believes the child to be nourished, not so much from the utero-placental circulation as from absorption of albumen from the amniotic fluid, the placenta acting simply as a respiratory organ.

---

We notice a constantly increasing number of favorable reports upon the success of Dr. Alexander's operation for shortening the wound ligaments in posterior displacements and prolapsus of the uterus. It looks to be a reasonable procedure, and it is to be hoped that its results will be permanent.

---

The *Glasgow Medical Journal* contains a report of the removal, by Dr. Patterson, of an exceedingly large stone from the bladder of a man. It measured, in its long circumference, ten and five-eighths inches, and in its short circumference eight and one-eighth inches. Its weight was fifteen ounces.

---

Dr. Giddings, of Maine, reports (*Medical Record*) an ovarian tumor developed within eighteen months in a woman ninety years old. He fitly queries: "Who has seen so old a person develop an ovarian tumor?"

In St. Mary's Hospital, Brooklyn, N. Y., there are eleven different departments. Roosevelt Hospital has but a single surgeon for the entire surgical work. Both hospitals claim to be successful.

---

Bellevue Hospital, of New York, like the Sisters of Charity Hospital, of Buffalo, has found it advisable to establish an emergency hospital in the down-town districts of the city.

